# C-demethylation and 1, 2-amino shift in (E)-2-(1-(3-aminophenyl) ethylidene)hydrazinecarboxamide to (E)-2-(2-aminobenzylidene)hydrazinecarboxamide and their applications

**DOI:** 10.1038/s41598-020-79027-1

**Published:** 2020-12-14

**Authors:** M. Sennappan, Sinosh Skariyachan, Praveen B. Managutti, Shubha Shridhar Gunaga

**Affiliations:** 1 Department of Chemistry, Dayananda Sagar College of Engineering, Bengaluru, 560078 India; 2 Department of Microbiology, St. Pius X College, Rajapuram, Kasaragod, Kerala 671532 India; 3 Department of Biotechnology, Dayananda Sagar College of Engineering, Bengaluru, 560078 India; 4 Solid State and Structural Chemistry Unit, Indian Institute of Science, Bengaluru, 560012 India

**Keywords:** Chemical biology, Computational chemistry

## Abstract

A Novel (E)-2-(1-(3-aminophenyl)ethylidene)hydrazinecarboxamide **1** was synthesized by traditional method and converted to (E)-2-(2-aminobenzylidene)hydrazinecarboxamide **2** by single step in DMSO at room temperature. Synthesized compound **1** was analysed by spectroscopy (NMR and LC–MS) techniques and molecule **2** was characterized using single crystal X-ray diffraction and spectroscopy (NMR and GC–MS) techniques. These analytical technique results revealed that, C-demethylation and 1, 2 amino shift in phenyl ring of compound **1** gives molecule **2**. DNA binding studies of compounds **1** and **2** was carried out by electronic absorption spectroscopy. This result revealed that, compounds **1** and **2** showed hyperchromism with bathochromic shift. Anticancer activity of compounds **1** and **2** is carried out by molecular docking with five receptors.Computer aided virtual screening demonstrated that the synthesized molecules possess ideal drug likeliness, pharmacokinetics features, toxicity profile for structure based drug discovery. The molecular docking studies revealed that the synthesized molecules are significant binding with the five selected cancer receptors with minimum binding energy (kcal/mol), number of hydrogen bonds, weak interaction, docking score and cluster RMS. The docking studies also suggested that the molecules showed interactions with DNA and the theoretical values of the binding are comparable with that of the experimental values. Hirshfeld surface analysis was used to analyze and quantify the intermolecular interactions in the crystal structure of compound **2**.

## Introduction

Semicarbazones are organic compounds possessing azomethine and urea functions, which are pharmacophores^1–4^. Semicarbazones have been extensively investigated owing to their potential applications in biological and pharmacological fields^5–7^. These are important classes of compounds which can coordinate with many metal ions because of the presence of donor atoms such as nitrogen and oxygen in their core structure^8–10^. In biological systems, C-demethylation occur in histones and DNA, catalysed by enzymes/chemical agents^11,12^. This is widely used in various therapeutic areas includes cancer, infectious diseases, inflammation, immunology, dermatology, epigenetics, psychiatric disease etc.^13–16^. O-demethylation is occurred in methyl ethers and methyl esters and N-demethylation in tertiary amines using harsh chemical reagents/conditions^17–21^. These chemical demethylation is used to make several valuable molecules like paroxetine, atanine, morphine etc.^22–24^. In biological systems, 1,2 amino shift is observed in aliphatic/aliphatic position of aromatic compounds which is catalyzed by aminomutases, B6 and B12^25^. The recent surveys and studies revealed that the synergistic effect of aromatic compounds significantly reduces the risk of cancer mortality. Anticancer drugs approved by FDA in 2018 are Vitrakvi, Lorbrena, Talzenna, Vizimpro, Akynzeo, Erleada, Lutathera etc.^26^. All are aromatic molecules possessing substitutents such as O, N, S, Cl, F etc. Substitutents are having lone pair of electrons which makes the molecules biologically and pharmacologically potent^27^. In contrast, platinum based chemotherapeutic agents are most widely used drugs in treatment of various cancers. Their remarkable success has been marred somehow because of several side effects such as nephrotoxicity, adverse effects on the peripheral nervous system as well as liver damage, severe emesis and drug resistant tumors etc.^28^. Limitations of platinum based drugs arouse to design novel organic molecules as anticancer drugs. DNA binding mechanism of molecules has been studied in order to develop anticancer drugs. Organic molecules interact with DNA helix structure through an intercalation/non-intercalation process intervene with the cell cycle such as translation, transcription and replication causing cell death^29^. In the light of above, detailed analyses of structure of novel synthesized semicarbazones are completed by different analytical techniques. The interaction mechanism of molecules with nucleic acid by electronic absorption spectroscopy were carried out. Anticancer activity of compounds **1** and **2** is carried out by molecular docking with five receptors and ideal drug likeliness, pharmacokinetics features, toxicity profile for structure based drug discovery of compounds **1** and **2** were evaluated using computer aided virtual screening. An estimation of close intermolecular interactions in the compound **2** is also reported using Hirshfeld surface analysis.

## Results and discussion

**Scheme 1:**
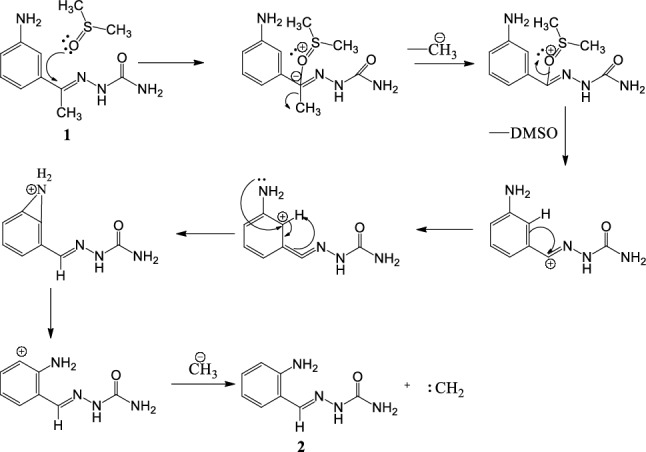
Mechanism of conversion of (E)-2-(1-(3-aminophenyl)ethylidene)hydrazinecarboxamide(**1**) to (E)-2-(2-aminobenzylidene)hydrazinecarboxamide(**2**).

The compound was kept for crystallization in polar solvent DMSO; the polar solvent attacked the carbonyl group of compound **1** gives carbanion as intermediate. The carbanion lost methyl group along with bonding electrons as methyl anion and also solvent regaining its original state given carbocation. The positive ion moves towards the phenyl ring and also shifting of proton from phenyl ring to azomethine carbon takes place. So that it produces positive charge on phenyl ring carbon, it is ortho position to amine group. However, 1, 2 shift of amino group in a phenyl ring and addition of hydride ion from methyl anion gave stable compound **2**. The intermediates of this conversion carbanion, carbocations and nitronium ion are stabilized by resonance, since molecule **1** has double bonds and heteroatoms in conjugation throughout the molecule. Conversion mechanism is given in Scheme 1.

### Single crystal X-ray diffraction studies

**Figure 1:**
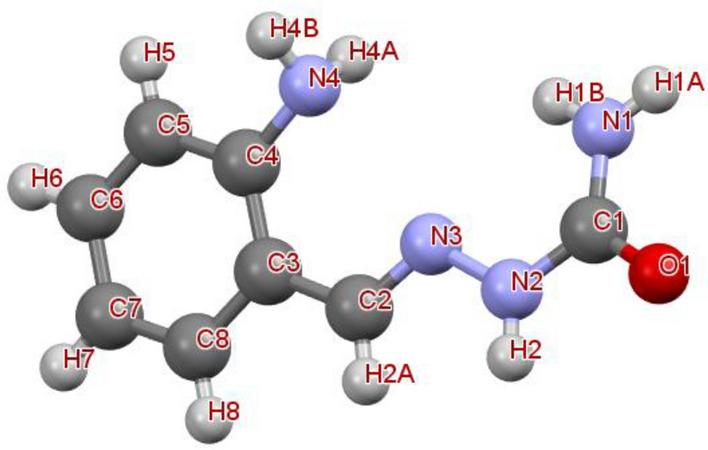
Structure of molecule 2 with labeling of atoms.

**Figure 2:**
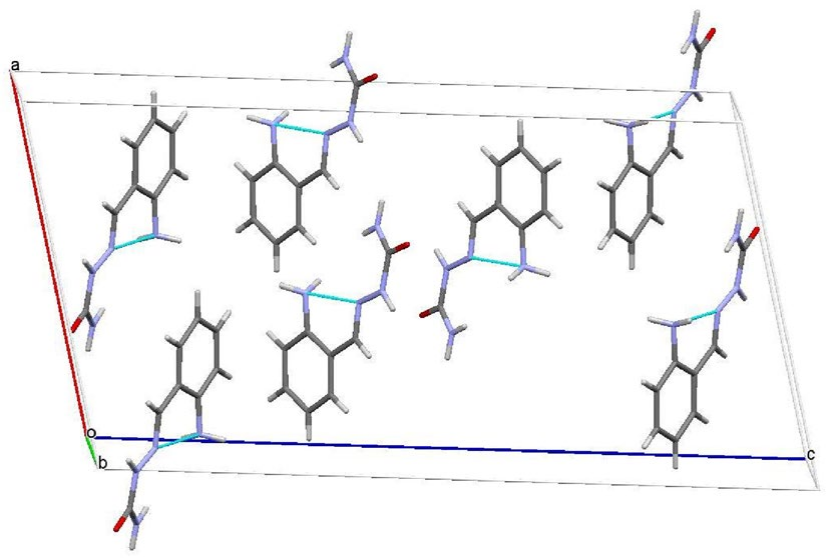
Unit cell packing diagram of molecule, 2.

**Figure 3:**
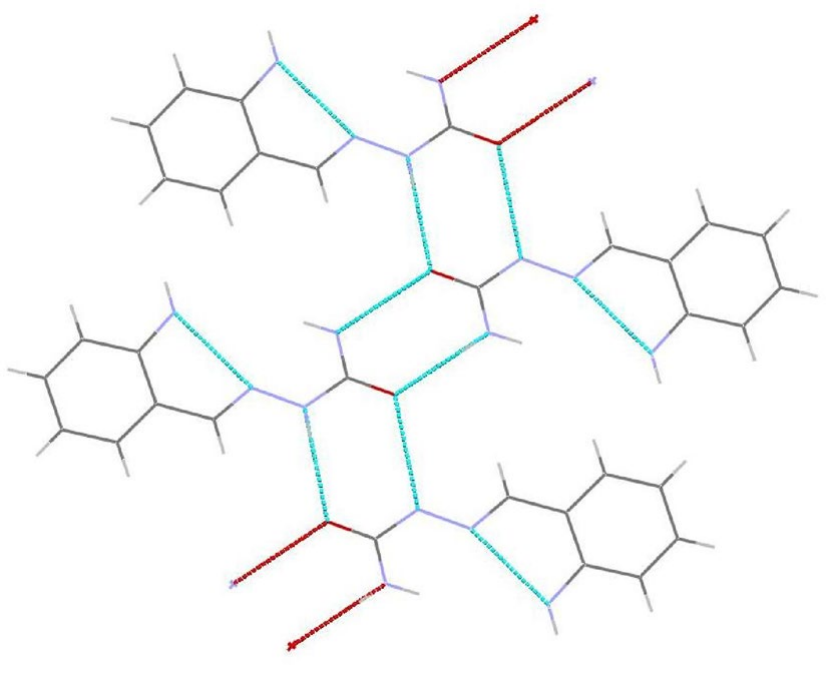
Structure of molecule 2 with hydrogen bonds.

The structure of molecule **2** with labeling of atoms, hydrogen bonds and packing diagram is given in Figs. 1, 2 and 3. Selected bond angles, bond lengths and hydrogen bonding data are listed in Tables 1 and 2. The C2-N3 bond length is 1.281(3) Å indicates that it is a characteristic of azomethine group^30^. Phenyl group in a molecule is delocalized bonding structure, confirms C–C bond lengths of ring in the ranging from 1.371(5) to 1.401(4) Å^31^. The bond length of C1-O1 is 1.250(3) Å; it is a characteristic of amide carbonyl group of semicarbazone^32^. The bond lengths of C4-N4 1.355(3) Å, N4-H4B 0.860(0) Å and N4-H4 0.860(1) Å, these bonds reveals that amino group attached to phenyl ring^33^. The conformation of molecule is stabilized by hydrogen bonds.

**Table 1:** Bond lengths and bond angles of compound **2.**

Atom	Atom	Length/Å	Atom	Atom	Atom	Angle/°
O1	C1	1.250(3)	C2	N3	N2	116.3(2)
N3	N2	1.373(3)	C1	N2	N3	121.3(2)
N3	C2	1.281(3)	C4	C3	C2	122.6(2)
N1	C1	1.329(3)	C4	C3	C8	118.4(2)
N2	C1	1.359(3)	C8	C3	C2	119.0(2)
N4	C4	1.355(3)	O1	C1	N1	123.1(2)
C3	C2	1.454(3)	O1	C1	N2	118.2(2)
C3	C4	1.391(4)	N1	C1	N2	118.7(2)
C3	C8	1.401(4)	N3	C2	C3	122.5(2)
C4	C5	1.389(4)	N4	C4	C3	121.1(2)
C8	C7	1.379(4)	N4	C4	C5	119.0(3)
C7	C6	1.381(5)	C5	C4	C3	119.9(3)
C6	C5	1.371(5)	C7	C8	C3	121.1(3)
N4	H4A	0.8600(0)	C8	C7	C6	119.6(3)
N4	H4B	0.8600( 1)	C5	C6	C7	120.2(3)
			C6	C5	C4	120.8(3)

**Table 2:** Hydrogen bond of the compound **2**.

D-H	d(D-H)	d(H..A)	< DHA	d(D..A)	A
N4-H4B	0.860	2.003	132.07	2.658	N3
N1-H1B	0.860	2.388	103.19	2.717	N3
N-H1A	0.860	2.027	172.55	2.882	O1
N1-H1B	0.860	2.825	119.25	3.331	O1
N2-H2	0.860	2.074	160.27	2.898	O1
C8-H8	0.930	2.804	130.39	3.480	N4
C7-H7	0.930	2.977	122.05	3.559	N4

### DNA binding studies

**Figure 4:**
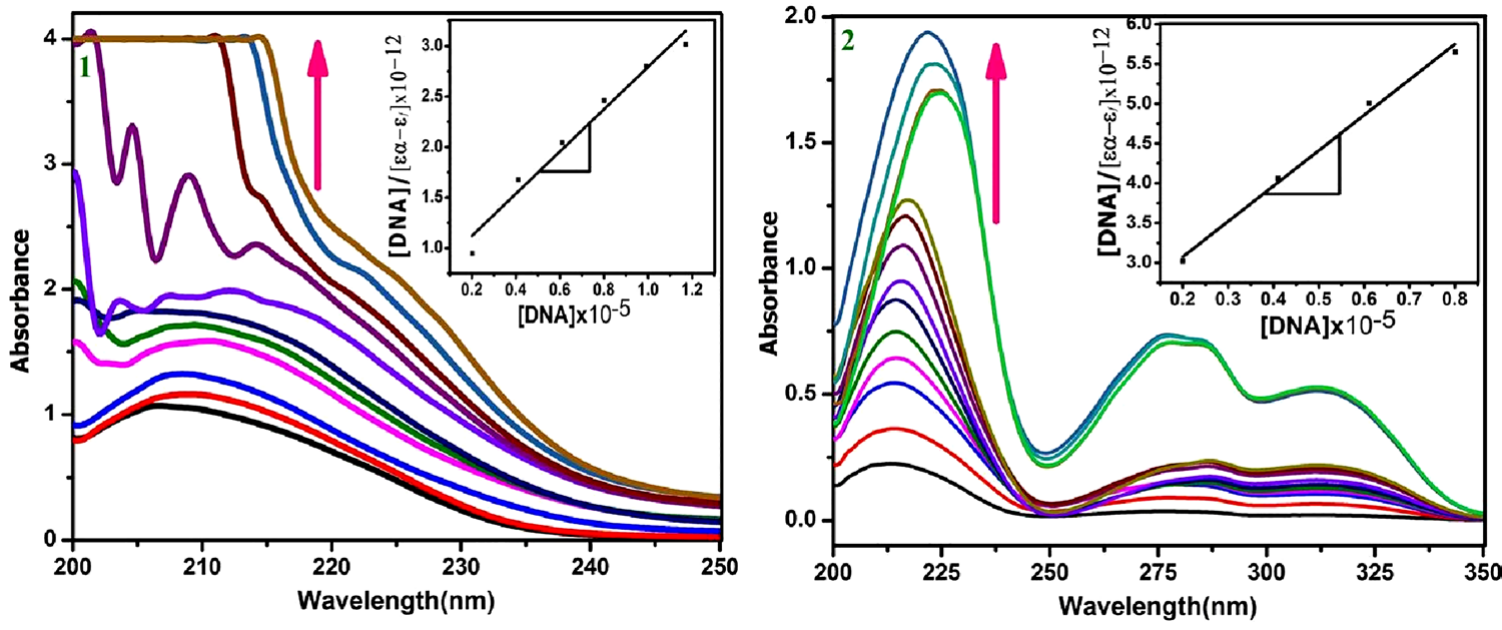
Electronic absorption spectra of molecule **1** and **2** are in the absence and presence of increasing amounts of CT-DNA (25µL—350µL). Arrow shows the increase absorbance with increase the DNA concentration. Inset: plot of [DNA]/ (εa-εf) Vs [DNA].

The electronic spectral data of compounds **1** and **2** were recorded in the absence and presence of rising amounts of CT-DNA (25 µL) (Fig. 4). The molecules were showed hyperchromism^34^ together with bathochromic shift. Hyperchromism with bathochromic shift could be the results of formation of covalent bond between the molecules and DNA; hence the combined forms are having more π-bonds and heteroatoms rather than molecules. The compound 1 showed hyperchromism of − 269.68% at ∆λ − 7.8; intrinsic DNA binding constant is 2.97 × 10^5^ M^−1^. Compound **2** displayed hyperchromism of − 652.21% at ∆λ − 11.4 and intrinsic DNA binding constant is 2.04 × 10^5^ M^−1^. The above results divulged that DNA binding ability is depending on position of amine group in the molecules. When it is in the meta position probably it is subjected to intermolecular hydrogen bond among the molecules and ortho position it is formed intramolecular hydrogen bond within the molecule.

### Computer aided virtual screening studies.

The predicted drug likeliness features of (E)-2-(1-(3-aminophenyl) ethylidene) hydrazine carboxamide and (E)-2-(2-aminobenzylidene) hydrazinecarboxamide are shown Table 3. From the table, it is clear that both the molecules are found to be suitable for various drugs like filters. The molecular refractivity, TPSA and Log Po/w and bioavailability score were in reliable cut off required for the computational screening. The filtering of the drug like features by various Cheminformatics filters showed that molecules are suitable for Ghose filter, Veber filter, Egan filter, Lipinski rule and CMC like rule. However, (E)-2-(2-aminobenzylidene) hydrazinecarboxamide showed violation of Lead like rule and Muegge filter. The drug like prediction by world drug index (WDI) like rule indicated that molecules are in 90% cutoff; it is ideal features for the lead molecules. The pharmacokinetic features of the two selected lead molecules are shown in Table 4. From the table, it is clear that, the two molecules showed almost similar pharmacokinetics features. Blood brain barrier permeability, Buffer solubility, human epithelial colorectal adenocarcinoma cell permeability, CYP inhibition, human intestinal absorption, plasma protein binding, pure water solubility and skin permeability models showed various values, signifies that most the pharmacokinetic features are in the acceptable range for ideal drug. The toxicity features of the selected lead molecules are shown in Table 5. The study indicated that, both molecules showed positive for carcinogenicity in mouse models and negative in rat models. Similarly, the compounds were predicted to be mutagens by Ames test. The predicted toxicity in algae and fish such as daphina, medaka and minnow were displayed varying results however, these results can be considered as features for ideal lead molecules. The in vitro Ames test results in TA100 strain were predicted to be negative for both the compounds. The in vitro hERG inhibition of compound **1** and compound **2** were predicted to be low risk and medium risk respectively. Thus, virtual screening and chemoinformatics features of the two compounds showed that these compounds probably possess ideal drug likeliness, pharmacokinetics and toxicity profile required to be considered the lead for structure based drug discovery.

**Table 3:** The drug likeliness features of the selected compounds predicted by PreADMET and SwissADME.

Compound	Molecular formula	IUPAC name	Molecular weight (g/mol)	Molar refractivity	Topological polar surface Area (TPSA) (Å^2^)	Log Po/w	Bioavailability score	Ghose filter	Veber filter	Egan filter	Muegge filter	Lipinski rule of five	CMC like rule	Lead like rule	MDDR like rule	WDI like rule
1	C_9_H_12_N_4O_	(E)-2-(1-(3-aminophenyl) ethylidene) hydrazinecarboxamide	192.22	55.21	93.50	0.43	0.55	Suitable	Suitable	Suitable	Suitable	Suitable	Qualified	Violated	Mid-structure	In 90% cutoff
2	C_8_H_10_N_4O_	(E)-2-(2-aminobenzylidene) hydrazinecarboxamide	178.19	50.40	93.50	0.33	0.55	Suitable	Suitable	Suitable	Violated	Suitable	Qualified	Violated	Mid-structure	In 90% cutoff

**Table 4:** The ADME features of the selected compounds predicted by PreADMET web server.

Compound	IUPAC Name	BBB (*C*_brain_*/C*_blood_)^a^	Buffer solubility (mg/L)	Caco2 (nm/s)^b^	CYP 2C19 inhibition CYP 2C9 inhibition CYP 2D6 inhibition CYP 3A4inhibition CYP 3A4 substrate Pgp inhibition	HIA^c^ (%)	MDCK (nm/s)^d^	PPB (%)^e^	Pure water solubility (mg/L)	Skin permeability (log kp, cm/h)^f^
1	(E)-2-(1-(3-aminophenyl) ethylidene) hydrazinecarboxamide	0.343929	2093.38	20.8564	Non	83.106555	106.93	7.319536	479.334	-3.97404
2	(E)-2-(2-aminobenzylidene) hydrazinecarboxamide	0.343711	24.1669	0.649922	Non	82.159306	50.2498	5.278023	1270.08	-3.95658

^a^In vivo blood brain–barrier penetration—(*C*_brain_*/C*_blood_) for high absorption to CNS > 2.0; middle adsorption to CNS: 2.0 ≈ 0.1; low absorption to CNS < 0.1.

^b^In vitro caco2 cell permeability—Low < 4; middle: 4–7; high > 7.

^c^ Human intestinal (HIA%) absorption—Poor: 0–20%; moderate: 20–70%; well: 70–100%.

^d^In vitro MDCK cell permeability—Low < 25; middle: 25–500; high > 500.

^e^In vitro plasma protein binding—weakly bound: < 90%; strongly bound: > 90%.

^f^In vitro skin permeability—Low < 1; middle: 1–2; high > 2.0.

**Table 5:** The toxicity features of the selected compounds predicted by PreADMET web server.

Compound	IUPAC Name	Acute algae toxicity	Acute daphina toxicity	Ames test	Carcinogenicity (Mouse)	Carcinogenicity (Rat)	in vitro hERG inhibition	Acute fish toxicity (medaka)	Acute fish toxicity (minnow)	in vitro Ames test result in TA100 strain
1	(E)-2-(1-(3-aminophenyl) ethylidene) hydrazinecarboxamide	0.22763	1.43559	Mutagen	Positive	Negative	Low risk	2.54523	1.70397	Negative
2	(E)-2-(2-aminobenzylidene) hydrazinecarboxamide	0.271746	1.21702	Mutagen	Positive	Negative	Medium risk	1.83596	1.34808	Negative

### Study of binding potential of compounds with various cancer targets

**Figure 5:**
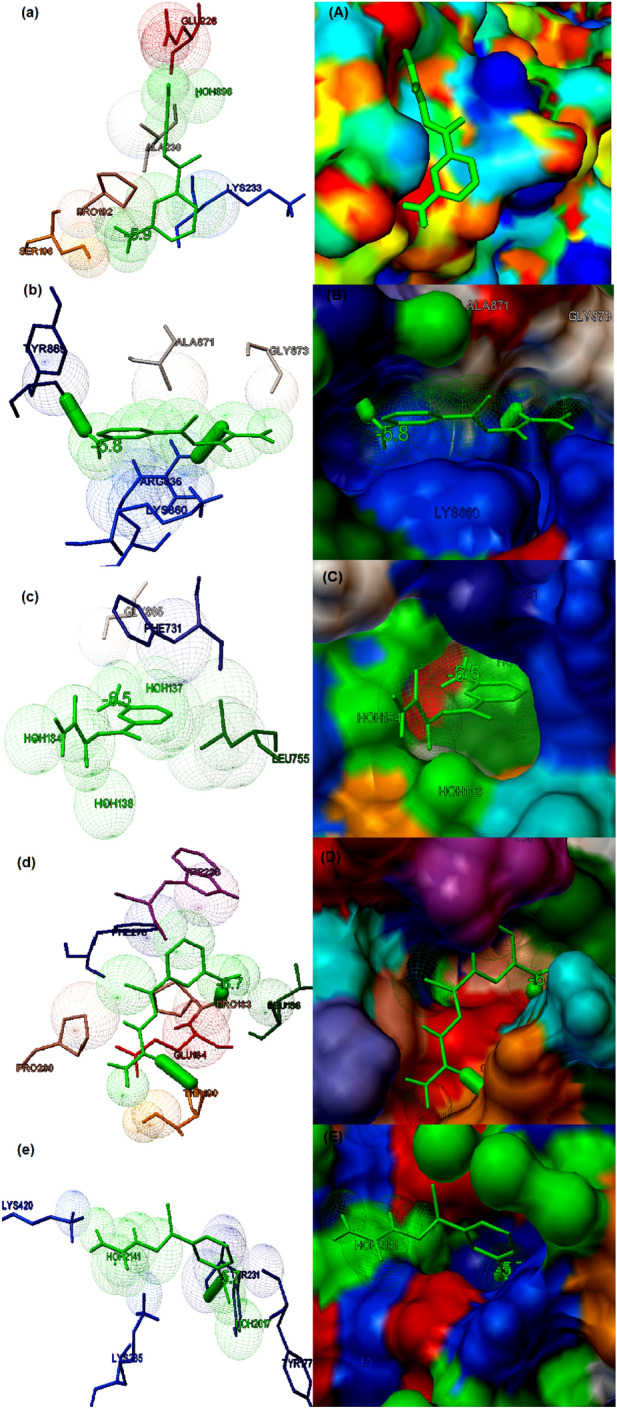
The binding potential of the compound **1** to the five selected cancer receptor studied by molecular docking (**a**) The best binding Interaction of (E)-2-(1-(3-aminophenyl) ethylidene) hydrazinecarboxamide and β-catenin predicted by molecular docking with binding energy of − 5.9 kcal/mol. The ligand is highlighted in green coloured stick figures. The amino acid residues present in the binding activity of the β-catenin receptor is also highlighted (**b**) The best binding Interaction of (E)-2-(1-(3-aminophenyl) ethylidene) hydrazinecarboxamide and Epidermal growth factor receptor (EGFR) with binding energy of − 5.8 kcal/mol. Thick green coloured stuck indicate hydrogen bonding. The ligand is highlighted in green coloured stick figures and the amino acid residues present in the binding activity of the EGFR receptor is also shown in the figure (**c**) The best binding Interaction of (E)-2-(1-(3-aminophenyl) ethylidene) hydrazinecarboxamide and Kinase domain of Human HER2 (erbB2) predicted by molecular docking with binding energy of − 6.5 kcal/mol. The ligand is highlighted in green coloured stick figures and the amino acid residues present in the binding activity of the EGFRK receptor are also highlighted. (**d**) The best binding Interaction of (E)-2-(1-(3-aminophenyl) ethylidene) hydrazinecarboxamide and Cyclin D1-cyclin-dependent kinase 4 with binding energy of − 6.5 kcal/mol. Thick green coloured stuck indicate hydrogen bonding. The ligand is highlighted in green coloured stick figures and the amino acids present in the binding activity of Cyclin D1-cyclin-dependent kinase 4 is also highlighted. (**e**) The best binding Interaction of (E)-2-(1-(3-aminophenyl) ethylidene) hydrazinecarboxamide and RAC-beta serine/threonine-protein kinase B with binding energy of − 6.5 kcal/mol. The ligand is highlighted in green coloured stick figures and the amino acids present in the binding activity of the RAC-beta serine/threonine-protein kinase B receptor is also shown in the figure. The part shown in uppercase indicate the molecular surface display of the best binding pose of the interaction between the compound **1** and selected cancer receptor.

Each of the three dimensional structures of the selected cancer protein targets (Table 6) were docked with the compounds **1** and **2**. The binding potential of these compounds towards the prioritized targets are shown in Table 7. From the docking studies, it is clear that each of the synthesized compounds possessed good binding potential towards five selected cancer targets with minimum binding energy (kcal/mol), cluster root mean square deviation, number of hydrogen bond stabilization and maximum number of interacting residues. The molecular docking studies clearly showed that the synthesized compound **1** is demonstrated good binding potential to all the five selected cancer targets. The compound showed good binding to β-catenin (PDB ID: 1JDH) with binding energy of − 5.9 kcal/mol. The main residues present in the binding cavity were found to be Ser106, Pro192, Glu228, Ala230 and Lys233 (Fig. 5a). The compound demonstrated the potential binding with epidermal growth factor receptor (EGFR) (PDB ID: 4R3P) with binding energy of − 5.8 kcal/mol. Arg836, Lys860, Tyr869, Ala871 and Gly873 were identified to be the main residues present in the binding cavity of the receptor. The interaction also stabilized by two hydrogen bond formation with the residues such as Arg836 and Tyr869 (Fig. 5b). Similarly, the compound exhibited good binding potential to kinase domain of human HER2 (ERBB2) (PDB ID: 3PP0) with binding energy of − 6.5 kcal/mol. The major residues present in the binding pose were identified to be Leu755, Gly865 and Phe731; however, no hydrogen bond involved in the interaction (Fig. 5c). The compound also showed potential binding to cyclin D1-cyclin-dependent kinase 4 (PDB ID: 2W96) with a binding energy of − 6.7 kcal/mol. The main residues present in the binding cavity were found to be Pro183, Leu186, Glu184, Thr190, Trp238, Phe278 and Pro280. The interaction also stabilized by two hydrogen bonds with the residues Pro183 and Thr190 (Fig. 5d). Similarly, compound **1** also demonstrated good binding with RAC-beta serine/threonine-protein kinase B (PDB ID: 1GZK) with a binding energy of − 5.7 kcal/mol. The interaction is stabilized by a hydrogen bond with Thy231 and the major residues involved in the binding were found to be Thr231, Tyr177, Lys285 and Lys420 (Fig. 5e).

**Table 6:** Selection of probable drug targets from various types of cancers for structure based drug screening.

Gene number	Gene name	Drug target (Gene products)	PDB ID	Type of cancer involved	References
1499	CTNNB1	*β*-catenin	1JDH	Gastric cancer, colorectal cancer, endometrial cancer, thyroid cancer, hepatocellular carcinoma	Graham et al.^35^
1956	EGFR	Epidermal growth factor receptor	4R3P	Oral cancer, esophageal cancer, gastric cancer, bladder cancer, choriocarcinoma, cervical cancer, glioma, laryngeal cancer	Park et al.^36^
2064	ERBB2	Kinase domain of human HER2 (erbB2)	3PP0	Gastric cancer, Pancreatic cancer, Bladder cancer, endometrial cancer, ovarian cancer, choriocarcinoma, cervical cancer, breast cancer, cholangiocarcinoma	Aertgeerts et al.^37^
595	CCNDI	Cyclin D1-cyclin-dependent kinase 4	2W96	Hairy cell leukemia, multiple myeloma, oral cancer, esophageal cancer, breast cancer, laryngeal cancer	Day et al.^38^
P31751	AKT2	RAC-beta serine/threonine-protein kinase B	1GZK	Lung cancer, neuroblastoma, gastric cancer	Yang et al.^39^

The drug targets were screened based on the virulent function in the metabolic pathways of each type of cancer. The structural coordinates of these drug targets were retrieved from PDB.

**Table 7:** The binding potential of (E)-2-(1-(3-aminophenyl) ethylidene) hydrazinecarboxamide and (E)-2-(2-aminobenzylidene) hydrazinecarboxamide towards selected drug targets of various types of cancers predicted by molecular docking studies by AutoDock Vina.

Cancer receptors and PDB ID	Ligand molecules	Interacting residues	Cluster RMS (Å)	No. of hydrogen bonds and interacting amino acids	Binding energy (kcal/mol)
*β*-catenin (PDB ID: 1JDH)	(E)-2-(1-(3-aminophenyl) ethylidene) hydrazinecarboxamide	Ser106, Pro192, Glu228, Ala230, Lys233	0.00	Nil	− 5.9
	(E)-2-(2-aminobenzylidene) hydrazinecarboxamide	Phe21, Lys22, Glu36, Val349, Lys354	0.00	Nil	− 5.4
Epidermal growth factor receptor (EGFR) (PDB ID: 4R3P)	(E)-2-(1-(3-aminophenyl) ethylidene) hydrazinecarboxamide	Arg836, Lys860, Tyr869, Ala871, Gly873	0.00	02 Arg836,Tyr869	− 5.8
	(E)-2-(2-aminobenzylidene) hydrazinecarboxamide	His393, Trp880, Lys879, Lys913, Gly917, Asp916	0.00	01 Asp916	− 5.7
Kinase domain of human HER2 (ERBB2) (PDB ID: 3PP0)	(E)-2-(1-(3-aminophenyl) ethylidene) hydrazinecarboxamide	Leu755, Gly865, Phe731	0.00	Nil	− 6.5
	(E)-2-(2-aminobenzylidene) hydrazinecarboxamide	Leu755, As758, Ala763, Glu766	0.00	01 Asn758	− 6.2
Cyclin D1-cyclin-dependent kinase 4 (PDB ID: 2W96)	(E)-2-(1-(3-aminophenyl) ethylidene) hydrazinecarboxamide	Pro183, Leu186, Glu184, Thr190, Trp238, Phe278, Pro280	0.00	02 Pro183,Thr190	− 6.7
	(E)-2-(2-aminobenzylidene) hydrazinecarboxamide	Glu184, Leu186, Gln188, Phe278, Pro280	0.00	Nil	− 6.0
RAC-beta serine/threonine-protein kinase B (PDB ID: 1GZK)	(E)-2-(1-(3-aminophenyl) ethylidene) hydrazinecarboxamide	Thr231, Tyr177, Lys285, Lys420	0.00	01 Thy231	− 5.7
	(E)-2-(2-aminobenzylidene) hydrazinecarboxamide	Glu236, Phe238, Glu279, Asp440	0.00	01 Asp440	− 5.4

**Figure 6:**
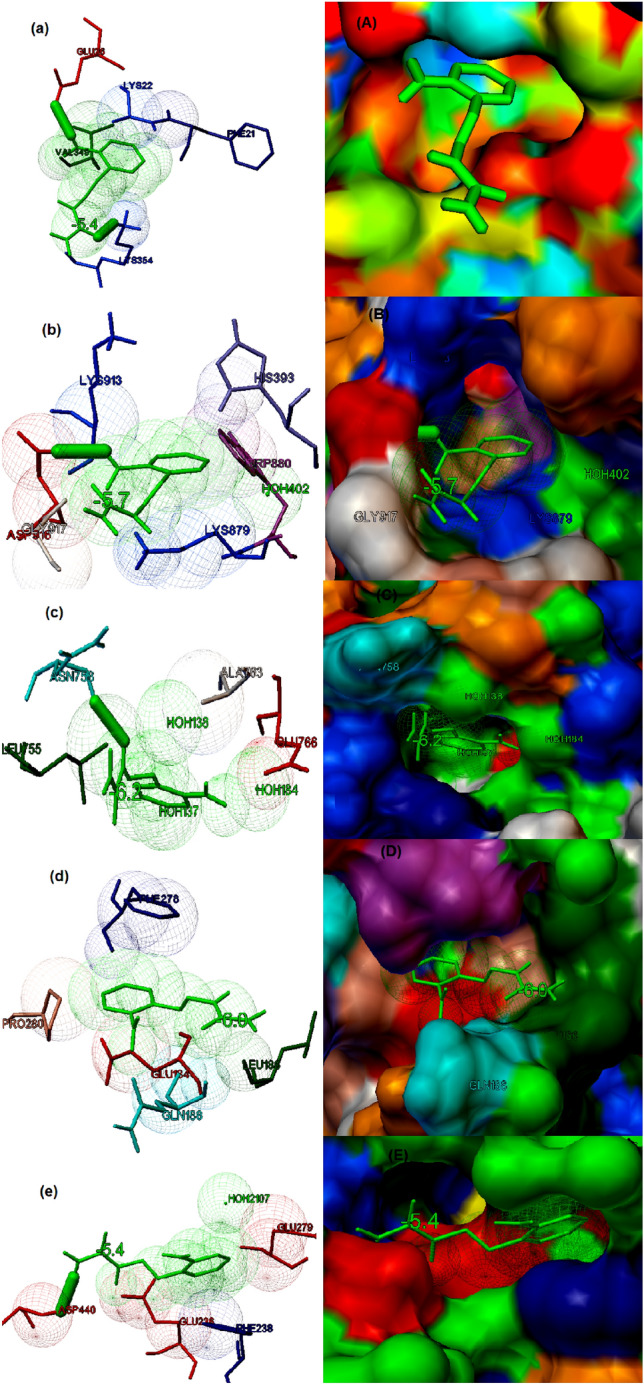
The binding potential of the compound **2** to the five selected cancer receptor studied by molecular docking (**a**) The best binding Interaction of (E)-2-(2-aminobenzylidene) hydrazinecarboxamide and β-catenin predicted by molecular docking with binding energy of − 5.9 kcal/mol. The ligand is highlighted in green coloured stick figures. The amino acid residues present in the binding activity of the β-catenin receptor is also highlighted (**b**) The best binding Interaction of (E)-2-(2-aminobenzylidene) hydrazinecarboxamide and Epidermal growth factor receptor (EGFR) with binding energy of − 5.7 kcal/mol. Thick green coloured stuck indicate hydrogen bonding. The ligand is highlighted in green coloured stick figures and the amino acid residues present in the binding activity of the EGFR receptor is also shown in the figure (**c**) The best binding Interaction of ((E)-2-(2-aminobenzylidene) hydrazinecarboxamide and Kinase domain of Human HER2 (erbB2) predicted by molecular docking with binding energy of − 6.2 kcal/mol. The ligand is highlighted in green coloured stick figures and the amino acid residues present in the binding activity of the EGFRK receptor are also highlighted. (**d**) The best binding Interaction of (E)-2-(2-aminobenzylidene) hydrazinecarboxamide and Cyclin D1-cyclin-dependent kinase 4 with binding energy of − 6.0 kcal/mol. Thick green coloured stuck indicate hydrogen bonding. The ligand is highlighted in green coloured stick figures and the amino acids present in the binding activity of Cyclin D1-cyclin-dependent kinase 4 is also highlighted. (**e**) The best binding Interaction of (E)-2-(2-aminobenzylidene) hydrazinecarboxamide and RAC-beta serine/threonine-protein kinase B with binding energy of − 5.4 kcal/mol. The ligand is highlighted in green coloured stick figures and the amino acids present in the binding activity of the RAC-beta serine/threonine-protein kinase B receptor is also shown in the figure. The part shown in uppercase indicate the molecular surface display of the best binding pose of the interaction between the compound **2** and selected cancer receptor.

The molecular docking studies also demonstrated that the synthesized compound **2** exhibited potential binding to all the five selected cancer targets. The compound showed a good binding to β-catenin (PDB ID: 1JDH) with binding energy of − 5.9 kcal/mol. The main residues present in the binding cavity were found to be Phe21, Lys22, Glu36, Val349 and Lys354 (Fig. 6a). The compound **2** demonstrated the potential binding with epidermal growth factor receptor (EGFR) (PDB ID: 4R3P) with binding energy of − 5.7 kcal/mol. The interaction is stabilized by a hydrogen bond which was formed with Asp916 and the major residues present in the binding cavity were identified to be His393, Trp880, Lys879, Lys913, Gly917 and Asp916 (Fig. 6b). Similarly, the compound **2** exhibited good binding potential to kinase domain of human HER2 (ERBB2) (PDB ID: 3PP0) with binding energy of − 6.2 kcal/mol. The major residues present in the binding pose are Leu755, As758, Ala763, Glu766 and the interaction is also stabilized by a hydrogen bond at Asn758 (Fig. 6c). The compound **2** exhibited potential binding to cyclin D1-cyclin-dependent kinase 4 (PDB ID: 2W96) with a binding energy of − 6.0 kcal/mol. The main residues present in the binding cavity were found to be Glu184, Leu186, Gln188, Phe278 and Pro280 (Fig. 6d). Similarly, compound 2 also demonstrated good binding with RAC-beta serine/threonine-protein kinase B (PDB ID: 1GZK) with a binding energy of − 5.4 kcal/mol. The interaction is stabilized by a hydrogen bond with Asp440 and the major residues involved in the binding were found to be Glu236, Phe238, Glu279, Asp440 (Fig. 6e).

**Figure 7:**
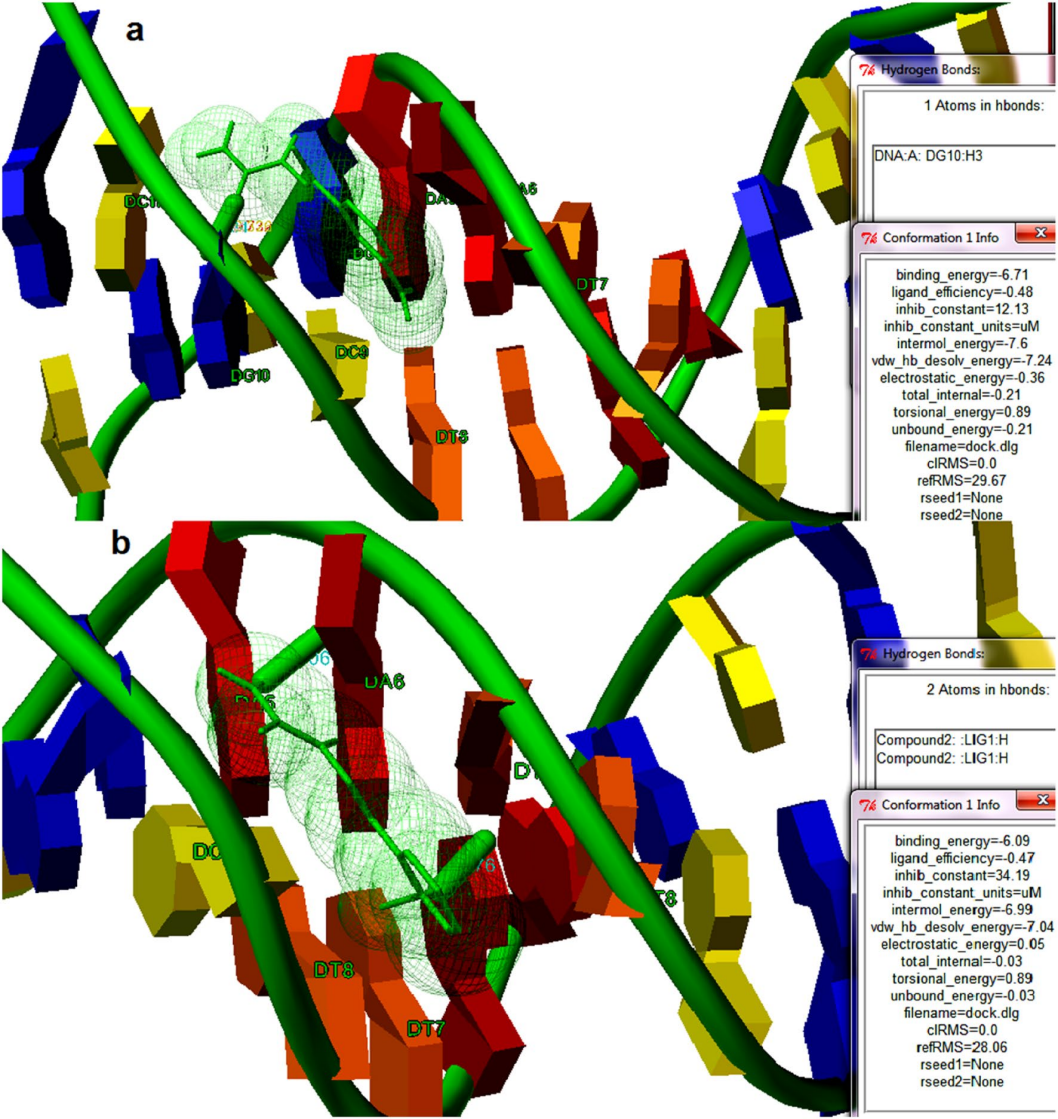
The binding potential of compounds **1** and **2** towards CT-DNA (PDB ID: 2DYW) is modeled by molecular docking. (**a**) The best binding conformation of (E)-2-(2-aminobenzylidene) hydrazinecarboxamide and DNA predicted by molecular docking with the binding energy of − 6.71 kcal/mol is shown in the figure. The ligand is highlighted in green colored stick figures and teh DNA is also shown. A thick green colored stick indicates hydrogen bonding. The residues present in the binding cavity of the DNA are also highlighted (**b**) The best binding conformation of (E)-2-(2-aminobenzylidene) hydrazinecarboxamide and DNA with the binding energy of − 6.09 kcal/mol are shown in the figure. The binding energy (kcal/mol), ligand efficiency, inhibition constants (µM), intermolecular energies are also shown in both the figures.

When the binding of two synthesized compounds was predicted against calf thymus (CT) DNA (PDB ID: 2DYW) by molecular docking studies, the modeling studies demonstrated that both the compounds showed profound binding towards the DNA. The compound **1** (1, 2-amino shift in (E)-2-(1-(3-aminophenyl) ethylidene) hydrazinecarboxamide) showed interaction with DNA at DG10, and the interaction stabilized with a hydrogen bond. The compound **1** interacted within the two strands of the DNA (Fig. 7a). The binding energy of the interaction is estimated to be − 6.71 kcal/mol. The ligand efficiency and intermolecular energy were found to be − 0.48 and − 7.24 kcal/mol respectively. The inhibition constant was found to be 12.13 µM. Similarly, when the binding of the compound 2 ((E)-2-(2-aminobenzylidene) hydrazinecarboxamide) towards the CT- DNA (PDB ID: 2DYW) were predicted by molecular docking studies showed significant binding with the DNA. The compound **2** showed profound binding with DNA with the binding energy of − 6.71 kcal/mol. The compound interacted within the two strands and the interaction stabilized with DG 6 and DG7 with two hydrogen bonds (Fig. 7b). The ligand efficiency and intermolecular energy were found to be − 0.47 and − 6.99 kcal/mol respectively. The inhibition constant was found to be 34.13 µM. From the interaction modeling by molecular docking, it is clear that both compound **1** and compound **2** interacted within both the strands of the DNA in almost the nearer binding sites. As shown in the experimental studies, the computational interaction modeling of the molecules and the DNA showed profound binding and the theoretical prediction is comparable with the experimental finding. The theoretical binding constants, inhibition constants, and binding energies are comparable with the experimental finding that showed a concurrent result. As shown in the experimental studies, computational modeling also suggested that the DNA binding potential of the compounds depends on the position of the amine group in the aromatic ring of the molecules.

The synthesized compounds **1** and **2** demonstrated good drug likeliness, pharmacokinetic features, ideal toxicity parameters and good binding with all the five selected cancer drug targets. β-catenin is involved in various types of cancers such as gastric cancer, colorectal cancer, endometrial cancer, thyroid cancer, hepatocellular carcinoma and studies suggested that it is one of the potential targets for drug development^40^. Both the compounds showed good binding with these targets. The binding energy of the interaction is the same and the interaction is stabilized by various amino acids. Epidermal growth factor receptor (EGFR) is one of the major cancer receptor, responsible for several types of cancer such as oral cancer, esophageal cancer, gastric cancer, bladder cancer, choriocarcinoma, cervical cancer, glioma and laryngeal cancer. The target can be prioritized as one of the potential cancer target^41^. Both the synthesized compounds were found to be equally potential as the binding energy is almost same. Kinase domain of human HER2 (ERBB2) is another important target has potential role in several types of cancer such as gastric cancer, pancreatic cancer, bladder cancer, endometrial cancer, ovarian cancer, choriocarcinoma, cervical cancer, breast cancer and cholangiocarcinoma and can be considered as one of the potential target for anti-cancerous compound development^42^. Cyclin D1-cyclin-dependent kinase 4 is one of the major types of the cancer receptor which involved in various types of cancer such as hairy cell leukemia, multiple myeloma, oral cancer, esophageal cancer, breast cancer and laryngeal cancer and can be considered as one of the promising drug targets^43^. Both the molecules demonstrated good binding potential with the cancer target. RAC-beta serine/threonine-protein kinase B is another important kind of cancer receptor considered as potential drug targets for lung cancer, neuroblastoma and gastric cancer. Both the compound found to be interacted with this cancer receptor and probably possess good inhibitory potential to the cancer target^44^. The present study suggested that compound **1** possesses better binding potential to most of the selected cancer receptor, thus the compound probably considered as potential inhibitors towards all the five cancer receptors. Compound **2** also showed comparable binding potential with stable interactions with minimum binding energy.

There are several studies suggested the anticancer properties of various synthesized derivatives of hydrazinecarboxamide^45–48^. To the best of our knowledge, this is the first study demonstrated the binding potential of the synthesized compounds **1** and **2** towards five selected targets which are considered as the major targets in various types of cancer. The anticancerous properties of the synthesized compounds were suggested based on computational virtual screening and molecular docking studies. Thus, molecular dynamic simulations studies can be performed to confirm the stability of the docked complexes of the synthesized compounds and prioritized cancer targets. Further, the efficiency and inhibitory potential of the synthesized compounds towards the prioritized targets needed to be tested at lower micro molar concentration by appropriate in vitro assays to validate the prediction. The finding in the current study surely provides significant insights for further experimentations and future investigations.

### Hirshfeld surfaces analysis

Hirshfeld surface analysis gives information on a crystal contained unique information on each molecule by dividing the crystal space in to non-overlapping molecular volume. Which allows us to excess intermolecular interactions. Here, dnorm is the parameter for normalized contact distance, which is based on (di), (de) and (r vdW) van der Waals radii of atom, which gives the equation as follows^49^,

$${\text{d}}_{{{\text{norm}}}} = \left( {{{{\text{d}}_{{\text{i}}} - {\text{r}}_{{\text{i}}}^{{{\text{vdw}}}} } \mathord{\left/ {\vphantom {{{\text{d}}_{{\text{i}}} - {\text{r}}_{{\text{i}}}^{{{\text{vdw}}}} } {{\text{r}}_{{\text{i}}}^{{{\text{vdw}}}} }}} \right. \kern-\nulldelimiterspace} {{\text{r}}_{{\text{i}}}^{{{\text{vdw}}}} }}} \right) + \left( {{{{\text{d}}_{{\text{e}}} - {\text{r}}_{{\text{e}}}^{{{\text{vdw}}}} } \mathord{\left/ {\vphantom {{{\text{d}}_{{\text{e}}} - {\text{r}}_{{\text{e}}}^{{{\text{vdw}}}} } {{\text{r}}_{{\text{e}}}^{{{\text{vdw}}}} }}} \right. \kern-\nulldelimiterspace} {{\text{r}}_{{\text{e}}}^{{{\text{vdw}}}} }}} \right)$$

**Figure 8:**
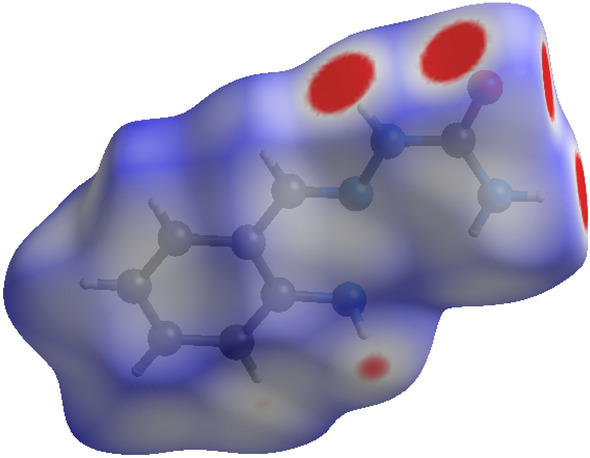
Dnorm mapped on Hirshfeld surface for visualizing the intercontacts of the compound. Color scaled in between − 0.10 au (blue) to 1.21 au (red).

**Figure 9:**
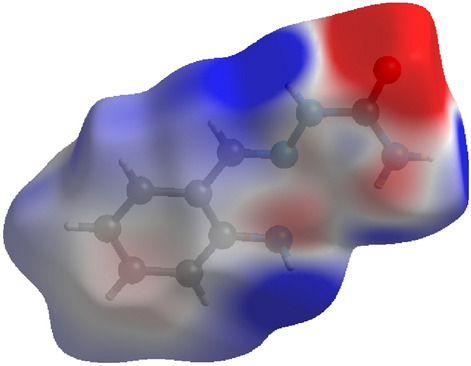
Electrostatic potential mapped on Hirsh field surface (different orientation) with ± 0.030 au. Blue region corresponds to positive electrostatic potential and red region to negative electrostatic potential.

**Figure 10:**
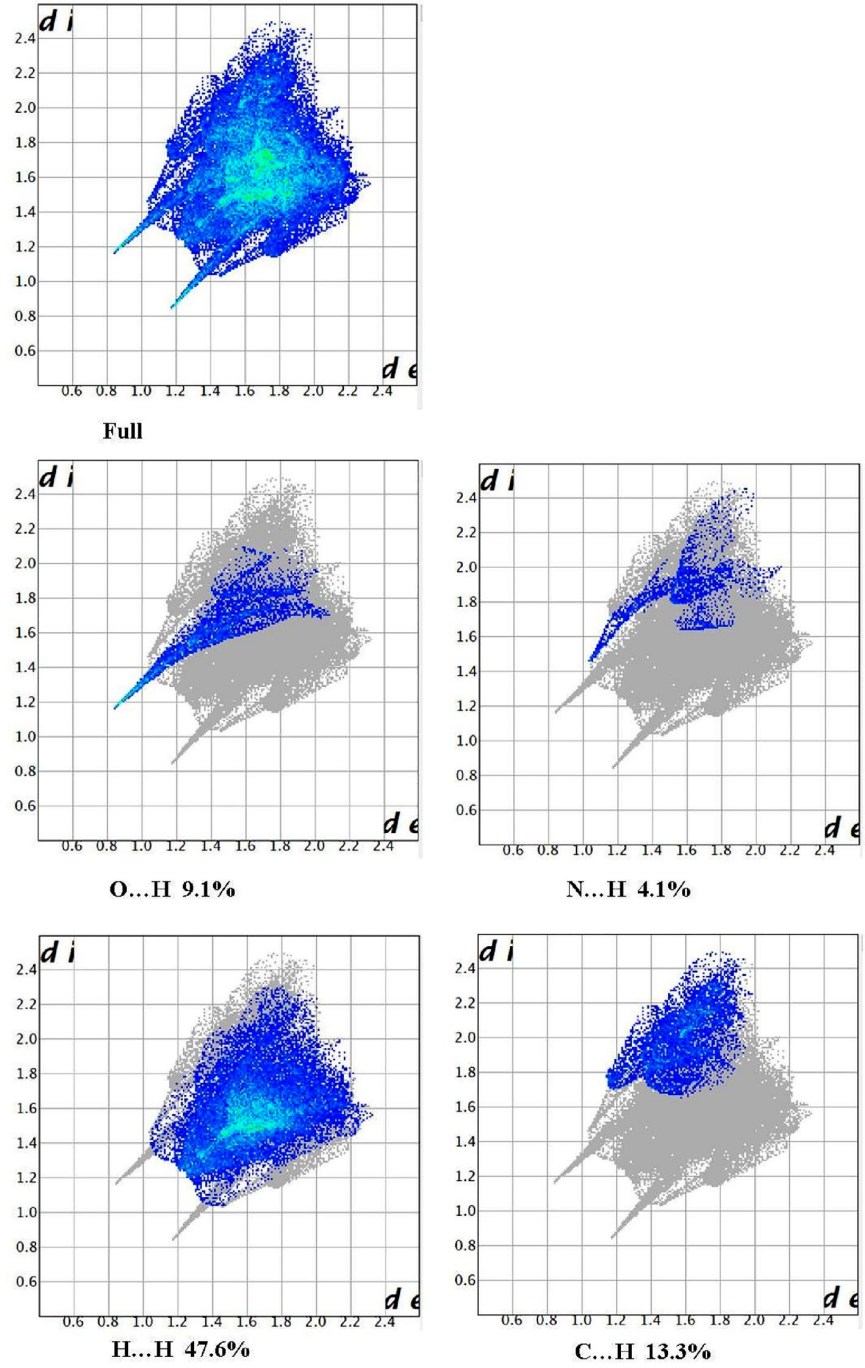
Fingerprint plots: Hirshfeld surfaces and 2D fingerprint plots of compounds and outline of the full fingerprint is shown in gray and other intercontacts from 2 to 10. di is the closest internal distance from a given point on the Hirshfeld surface and de is the closest external contacts.

The Hirshfeld anslysis resolves and quantifies the intercontact between atoms in the crystal structure Fig. 8. The red spots over the Hirshfeld surface images shows the involved intercontact in the intermolecular interactions^50^. The strong hydrogen bond spots as dark red region which is the result of short intercontact between the atoms showed on the dnorm surface and light red spots indicated the other intermolecular contact between the atoms. The electrostatic potential range is between plotted in − 0.030 a.u. to + 0.030 a.u on Hirshfeld surfaces based on Hartree–Fock theory and which is showed electrostatic potential over the Hirshfeld surfaces in Fig. 9 red region (hydrogen acceptor) negative. And blue region (hydrogen donor) positive electrostatic potentials^51^. The intercontacts with respect to di and de plotted for 2D fingerprint plots and quantified by using visualization of the Hirshfeld surfaces (Fig. 10). The intercontacts were found to be C…H (13.3%), H…H (47.6%), N…H (4.1%), O…H (9.1%). The intercontacts generated from the 2D Finger print plots are shown in Fig. 10. The major contributions of intercontacts Hirshfeld analysis are from H…H, C…H, O…H and S…H when compared to other intercontacts.

## Conclusion

The C-demethylation and 1,2 amino shift in phenyl ring takes place simultaneously in compound **1** under mild conditions and became molecule **2**. These kind of chemical changes was not reported in a compound to date. Furthermore, compound **2** cannot be made directly from 2-amino benzaldehyde, which has less stability; it is due to presence of aldehyde and amino group in the same molecule. Hence, in future this kind of easy chemical conversion can be used to make novel molecules and might be used in various fields. Nucleic acid interactions of compounds with CT-DNA was carried out, results revealed that they have displayed hyperchromism with bathochromic shift. Generally, this kind of results obtained from metal complexes has positively charged metal ions with nucleic acid gave electrostatic interaction. But these organic molecules **1** and **2** displayed excellent interaction with DNA and the molecular basis of the interaction was predicted by molecular docking studies. The computational virtual screening suggested that synthesized compounds possessed ideal drug likeliness, pharmacokinetic features and toxicity properties and demonstrated profound binding with selected cancer receptors are β-catenin, epidermal growth factor receptor, kinase domain of human HER2, cyclin D1-cyclin-dependent kinase 4 and RAC-beta serine/threonine-protein kinase B. Thus, biological studies of compounds provide ample foundation in the scale up of the applied approaches and nucleic acid interactions and computational prediction probably provides profound scope and application for the screening and designing of novel anticancer lead molecules. The Hirshfeld surface analysis of compound **2** was carried out and fingerprint plots were studied, results revealed that nature of molecular interactions and their contributions to the molecular surface.

## Experimental section

### Material and methods

All the reagents have been commercially obtained and have been used without further purification. Semicarbazide hydrochloride (99%) and 3-aminoacetophenone (97%) were purchased from Sigma-Aldrich, St. Louis, Missouri, United States. Sodium acetate trihydrate (99.5%), N, N-Dimethylformamide (99.9%), Dimethyl sulfoxide (99.8%) and methanol (99.5%) were obtained from Merck, Kenilworth, New Jersey, United States.

Melting point of the compounds was recorded using capillary tube in Sigma melting point apparatus, Sigma instruments. ^1^H-NMR spectrum obtained in DMSO-d6 using tetramethylsilane (TMS) on Advanced 200.13 MHz NMR spectrometer. ^13^C-NMR spectrum was obtained in DMSO-d6/CDCl_3_ using Bruker-300 MHz NMR. Single X-ray diffraction data was recorded on a Bruker Kappa Apex2 CCD diffractometer. The LC–MS of the compound **1** was recorded on Thermo, LCQ Deca XP MAX. GC–MS of molecule **2** was collected by Xevo G2-XS QT.

### Synthesis of (E)-2-(1-(3-aminophenyl)ethylidene)hydrazinecarboxamide(1)

**Scheme 2:**
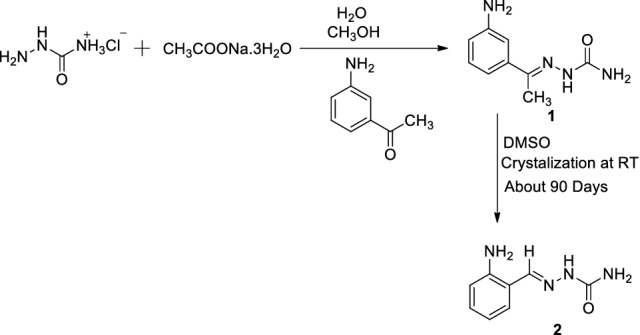
Synthesis of the molecule **1** and conversion of compound **1** to **2**.

The mixture of semicarbazide hydrochloride [1.11 g, 0.01 m] and sodium acetate trihydrate [1.63 g, 0.012 m] in water stirred for 15 min, added methanol containing 3-aminoacetophenone [1.35 g, 0.01 m] continued 10 min stirring. The colourless precipitate was filtered, washed with cold methanol and air dried. (Scheme 2). Yield: 78.0%, M.P: 166 °C, ^1^H-NMR (200.12 MHz, DMSO-d6, ppm) δ: 2.19 (s, 3H, CH_3_), 2.59 (s, 2H, NH_2_), 3.08 (s, DMSO), 4.20 (s, 2H, CONH_2_), 6.67–7.14 (m, 4H, Ar), 8.72 (s, 1H, NH). ^13^C NMR (300MHZ, DMSO-d6 and CDCl_3_, ppm) δ: 156.64 (1C, C-NH_2_), 146.42 (1C, C=O), 144.26 (1C, C = N) 137.91–110.72 (5C, Ar), 12.31 (1C, CH_3_), 77.43–76.56 (CDCl_3_), 39.57–37.87 (DMSO).LC–MS (m/z): 193.09 [M + 1].

### Conversion of (E)-2-(1-(3-aminophenyl)ethylidene)hydrazinecarboxamide(1) to (E)-2-(2-aminobenzylidene)hydrazinecarboxamide(2)

(E)-2-(1-(3-aminophenyl)ethylidene)hydrazinecarboxamide (Scheme 2) is dissolved in DMF/DMSO, kept at room temperature about 90 days. The formed dark brown crystals are separated out, washed with water and air dried. M.P: 231 °C. ^1^H-NMR (200.12 MHz, DMSO-d6, ppm) δ: 3.61 (s, DMSO), 6.37 (s, 2H, CONH_2_), 6.79–7.69 (m, 4H, Ar), 8.12 (s, 1H, CH = N), 10.18 (s, 1H, NH). ^13^C NMR (300MHZ, DMSO-d6 and CDCl_3_, ppm) δ: 154.82 (1C, C-NH_2_), 154.09 (1C, C = O), 136.75 (1C, C = N), 128.47- 114.23 (5C, Ar), 77.43–76.55 (CDCl_3_), 38.43–36.76 (DMSO).GC–MS (m/z): 202 [M + 1Na].

### X-ray crystallography

The compound **1** was dissolved in polar solvent DMSO and kept for crystallization at room temperature for three months. The colourless solution of compound **1** was slowly changed to dark brown and gave dark brown colour crystals, it is compound **2**. A specimen of crystal size 0.2 × 0.17 × 0.15 mm^3^ was selected for data collection on Bruker Kappa Apex2 CCD diffractometer at 296(2) K. Single crystal X-ray diffractometer equipped with graphite monochromated MoKα (λ = 0.71073) radiation, operated at 40KV and 30 mA. The structure of molecule **2** was solved by direct methods with the olex2.solve^52^ and refined with the ShelXL^53,54^. The crystallographic data of compound **2** are summarized in Table 8.

**Table 8:** Crystal data and structure refinement of compound **2.**

Identification code	NSC
CCDC No	1864018
Empirical formula	C_8_H_10_N_4_O
Formula weight	178.20
Temperature	296 K
Crystal system	monoclinic
Space group	C2/c
a	13.2228(10) Å
b	5.2826(4) Å
c	24.889(3) Å
Α	90°
β	104.092(8) °
γ	90°
Volume	1686.2(3) Å^3^
Z	8
ρ_calc_g	1.404 cm^3^
μ	0.099 mm^−1^
F(000)	752.0
Crystal size	0.2 × 0.17 × 0.15 mm^3^
Radiation	MoKα (λ = 0.71073)
2Θ range for data collection	6.752 to 49.984°
Index ranges	− 15 ≤ h ≤ 15, − 5 ≤ k ≤ 6, − 29 ≤ l ≤ 29
Reflections collected	5254
Independent reflections	1491 [R_int_ = 0.0387, R_sigma_ = 0.0279]
Data/restraints/parameters	1491/0/119
Goodness-of-fit on F^2^	1.089
Final R indexes [I > = 2σ (I)]	R_1_ = 0.0698, wR_2_ = 0.2087
Final R indexes [all data]	R_1_ = 0.0776, wR_2_ = 0.2189
Largest diff. peak/hole	0.36/− 0.47 e Å^−3^

### DNA binding

DNA binding ability of organic molecules **1** and **2** in DMSO are examined in 50 mM Tris–HCl/NaCl buffer solution at pH 7.0. Molar absorbance coefficient of CT-DNA (6600 dm^3^ mol^−1^ cm^−1^) at 260 nm was used to calculate the concentration of nucleic acid^55^. The CT-DNA in buffer solution gave a ratio of absorbance at 260 and 280 nm (A260/A280) is 1.83, divulged that protein free nature of DNA^55^. Electronic absorption spectroscopic titrations were performed varying the concentration of nucleic acid from 0 to 2.5 × 10^−5^ M, while molecules remained constant (0.01 M). After equilibrium was reached (ca.5 min), the spectra were recorded against blank solution containing the same concentration of CT-DNA. The intrinsic binding constant (K_b_) of molecules was calculated using following equation^56^.

$$\left[ {{\text{DNA}}} \right]/\left( {\varepsilon {\text{a}} - \varepsilon {\text{b}}} \right) = \left[ {{\text{DNA}}} \right]/\left( {\varepsilon {\text{b}} - \varepsilon {\text{f}}} \right) + 1/{\text{Kb}}\left( {\varepsilon {\text{b}} - \varepsilon {\text{f}}} \right)$$

where [DNA] is the concentration of CT-DNA, εa, εb and εf correspond to apparent, bound and free extinction coefficients of compounds **1** and **2**. A plot of [DNA]/(εa − εf) Vs [DNA] gave a slope of 1/ (εb − εf) and a Y-intercept equal to 1/Kb (εb − εf), Kb is the ratio of the slope to the Y-intercept.

### Bioinformatics

#### Selection of probable drug targets of various types of cancer

Based on the extensive literature survey, the major targets involved in various types of cancer were identified. The description of the cancer drug targets selected in the present study is shown in Table 6. The selected cancer receptors are β-catenin (PDB: 1JDH), Epidermal growth factor receptor (PDB: 4R3P), Kinase domain of human HER2 (PDB: 3PP0), Cyclin D1-cyclin-dependent kinase 4 (PDB: 2W96) and RAC-beta serine/threonine-protein kinase B (PDB: 1GZK).

#### Preparation of proteins

Three dimensional structure of the selected receptors such as β-catenin (PDB: 1JDH)^35^, Epidermal growth factor receptor (PDB: 4R3P)^36^, Kinase domain of human HER2 (PDB: 3PP0)^37^, Cyclin D1-cyclin-dependent kinase 4 (PDB: 2W96)^38^ and RAC-beta serine/threonine-protein kinase B (PDB: 1GZK)^39^ were retrieved from Protein Data Bank. The coordinate files were analyzed and edited by adding polar hydrogen atoms with the help of AutoDock Tools^57^. The binding cavities present in the selected receptors were predicted by Q-SiteFinder^58^ and DEPTH server^59^. The structures were saved in pdbqt format.

#### Preparation of structure of ligands

The 2D structure of these ligands (Scheme 1) was converted in to 3D structures and structure optimization was carried out by ACD 3D viewer^60^. The 3D structure of these ligands was saved in pdb format. The ligand structure was loaded into MGL Tools and the root atoms, number of torsion, rotatable and nonrotatable bonds were assigned and the file was saved in pdbqt format for docking studies.

#### Computer aided virtual screening of the ligand molecules

The drug likeliness features of (E)-2-(1-(3-aminophenyl) ethylidene) hydrazinecarboxamide and (E)-2-(2-aminobenzylidene) hydrazinecarboxamide were analyzed by the computational tools such as PreADMET^61^ and SwissADME^62^. The main filters used for the drug likeliness are Lipinski’s rule of five, CMC-like rule, Lead-like rule, MDDR-like rule and WDI-like rule available in PreADMET^63–66^ and Ghose filter, Veber filter, Egan filter and Muegge filter were available in SwissADME. The molar refractivity, Log P, Topological polar surface area (TPSA) and bioavailability score were also predicted by SwissADME. Further, the adsorption, distribution, metabolism and excretion (ADME) features of these lead molecules by various statistical models available in PreADMET such as blood brain barrier (BBB) penetration, buffer solubility, human intestinal absorption (HIA), heterogeneous human epithelial colorectal adenocarcinoma (caco2) cell permeability, Madin Darby canine kidney (MDCK) cell permeability, plasma protein binding, CYP2C19 inhibition, CYP 2C9 inhibition, CYP2D6 inhibition, CYP3A4 inhibition, CYP3A4 substrate, Pgp inhibition, pure water solubility and skin permeability assays. The toxicity of the ligands was predicted by various options available in PreADMET such as acute algae toxicity, carcinogenicity in mouse rat models, in vitro hERG inhibition, acute fish toxicities in daphina, medaka and minnow models and in vitro Ames test.

### Molecular docking studies

#### Interaction modelling of the synthesized compounds and probable cancer targets

The binding potential of (E)-2-(1-(3-aminophenyl) ethylidene) hydrazinecarboxamide and (E)-2-(2-aminobenzylidene) hydrazinecarboxamide to the selected cancer receptors were studied by molecular docking by AutoDock Vina^67^. The grid box was assigned for each cancer target by setting the 3D coordinates for the binding pocket predicted by various binding pocket prediction server mentioned previously. The x, y, z coordinates for binding pockets in each target were assigned in the configuration file. The total grid data per map for each of the selected receptor was assigned by fixing the values for number of points in x, y, z-dimensions (size_x, size_y, size_z). Further, the parameter values for center grid box of each receptor (center_x, center_y, center_z) were assigned as per the standard protocol. The values for exhaustiveness in the docking simulation were assigned as per standard (Trott and Olson 2010). The molecular docking simulation was performed through command prompt. The output (log) files of the best nine confirmations were generated and conformations were ranked according to minimum binding energy (kcal/mol), docking score, cluster RMSD, number of hydrogen bond formed and the interacting residues present at the close proximity of 1.0 Å VWD scaling factor. Out of which, the first confirmations were selected as the best predicted model. The receptor ligand complex was visualized in MGL tools and PyMol^68^.

#### Interaction modeling of the synthesized compounds and CT-DNA

The binding potential of (E)-2-(1-(3-aminophenyl) ethylidene) hydrazinecarboxamide and (E)-2-(2-aminobenzylidene) hydrazinecarboxamide towards the CT- DNA was predicted by molecular docking studies. The 3D structure of the DNA (PDB ID: 2DYW) was retrieved from the PDB database. The structure was analysed thoroughly and the bound form of the ligand was removed and the targets were prepared for docking studies as per the standard protocol^68^. The 3D structure of each compound was loaded in AutoDock tool and the ligands were prepared by setting the root atom, the number of torsions, and other parameters for the ligand preparation as per the standard protocol^68^. The binding sites were analysed by AutoGrid program and the grid maps were generated. The molecular docking was performed by AutoDock program using Lamarckian Genetic algorithm and the dock parameter files were generated. The best-docked conformations were analysed based on the binding energy (kcal/mol), number of hydrogen bonds, and other weak interactions in MGL tool. The binding and inhibition constant (µM), and binding energy were also estimated, and the theoretical values were compared with that of the experimental binding values.

### Hirshfeld surface analysis

The Hirshfeld surfaces computational program used to create the graphical representations and is used to perform and quantify the intermolecular interactions in terms of surface contribution by using the program Crystal Explorer 17.05^69–71^ and to generating electrostatic potential^72^ with TONTO^49^. This program explores molecular packing and provides surface information on different types of intermolecular interactions of the crystal and can be identified and their contribution of individual contact by2D fingerprint plots (FP) shown in the colour plot.

## Supplementary Information

Supplementary Information.
