# Supplementary material for: C-demethylation and 1, 2-amino shift in (E)-2-(1-(3-aminophenyl) ethylidene)hydrazinecarboxamide to (E)-2-(2-aminobenzylidene)hydrazinecarboxamide and their applications
Source: Sci Rep. 2020 Dec 14;10:21913. doi: 10.1038/s41598-020-79027-1 (PMC7736590; doi:10.1038/s41598-020-79027-1)
Supplement: Supplementary file 1 — Supplementary Information. [file 41598_2020_79027_MOESM1_ESM.docx]

**C-demethylation and 1, 2-amino shift in (E)-2-(1-(3-aminophenyl) ethylidene)hydrazinecarboxamide to (E)-2-(2-aminobenzylidene)hydrazinecarboxamide**

**and their Applications**

M. Sennappan^a^*, Sinosh Skariyachan^b,c^, Praveen B. Managutti^d^, Shubha Shridhar Gunaga^d^

^a^Department of Chemistry, Dayananda Sagar College of Engineering, Bengaluru 560078, India

^b^Department of Microbiology, St. Pius X College, Rajapuram, Kasaragod, Kerala 671532, India

^c^Department of Biotechnology, Dayananda Sagar College of Engineering, Bengaluru 560078, India

^d^Solid State and Structural Chemistry Unit, Indian Institute of Science, Bengaluru-560012, India

**
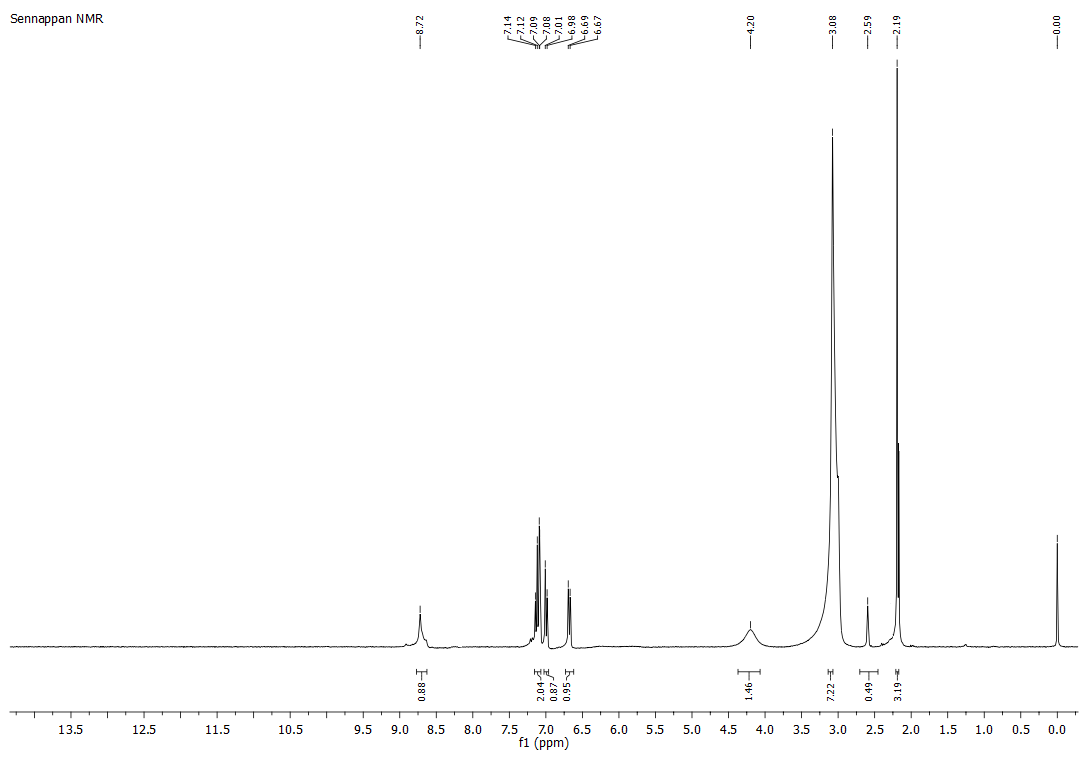
**

**Figure S1.** ^1^H NMR spectrum of compound **1.**


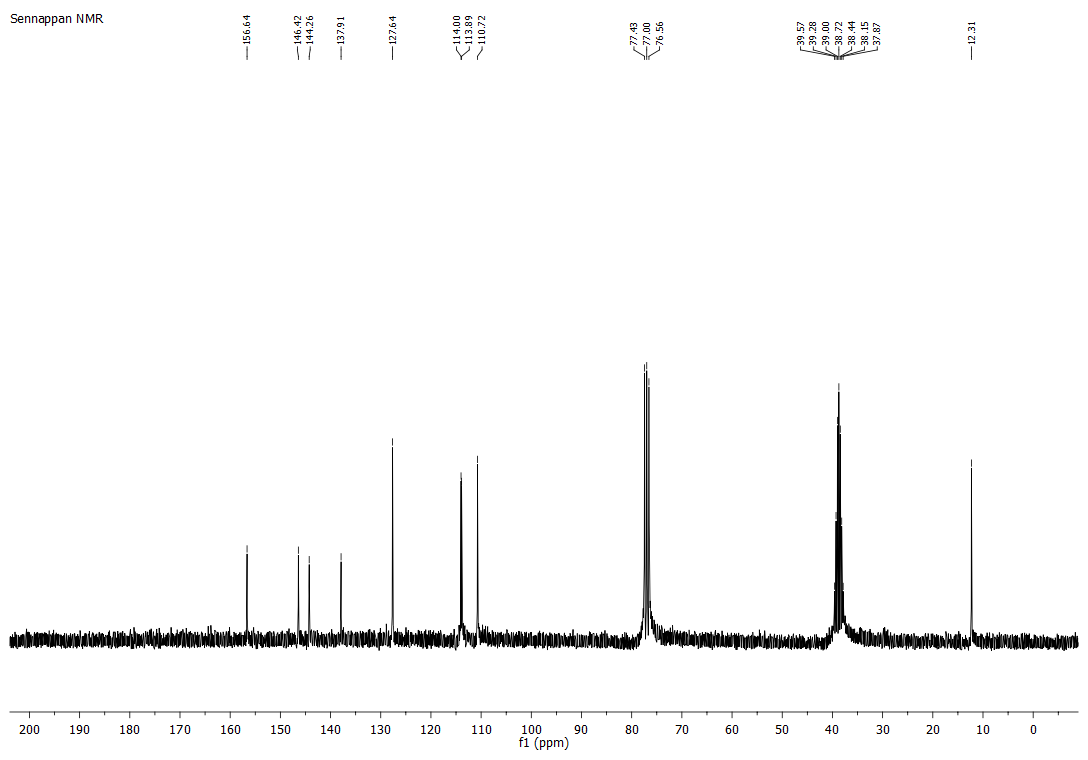


**Figure S2.** ^13^C NMR spectrum of compound **1.**

**
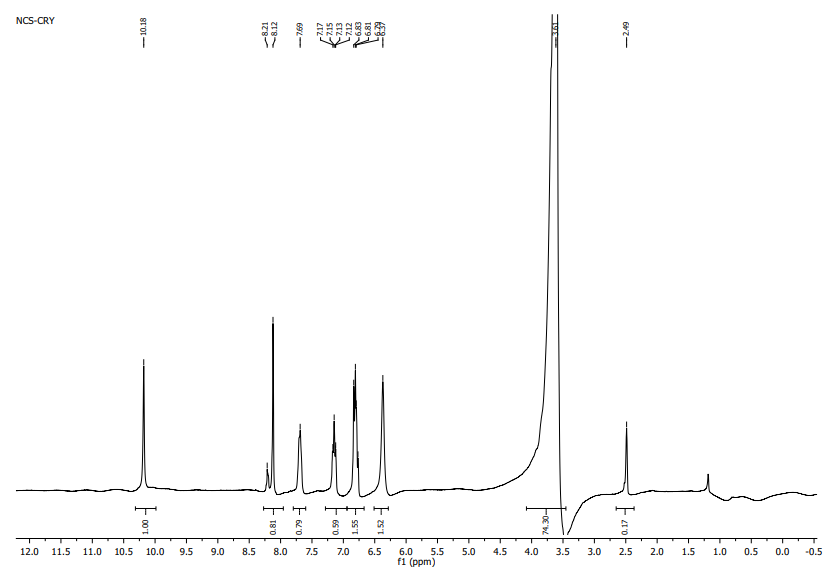
Figure S3.** ^1^H NMR spectrum of compound **2.
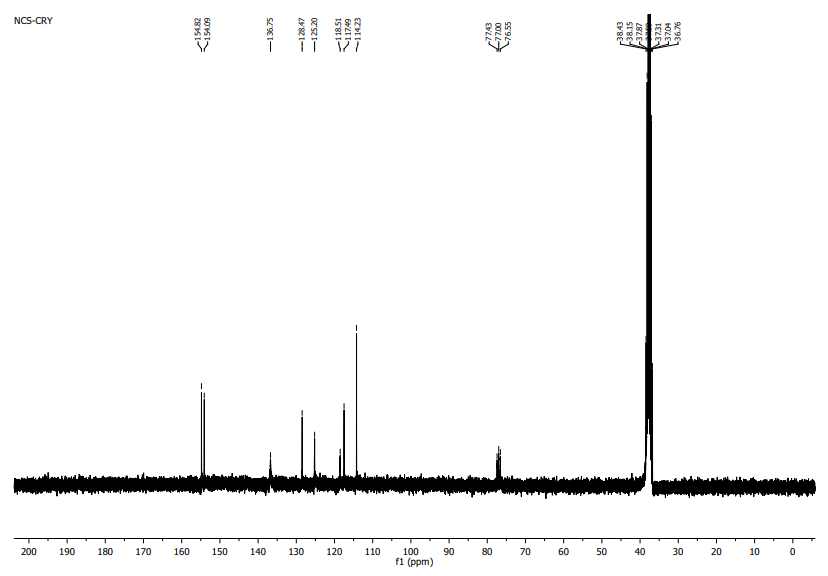
**

**Figure S4.** ^13^C NMR spectrum of compound **2.**

**Figure S5.** LC-MS of compound **1.**

**Figure S6.** GC-MS of compound **2.**
